# Artificial neural networks optimize the establishment of a Brazilian germplasm core collection of winter squash (*Cucurbita moschata* D.)

**DOI:** 10.1038/s41598-024-54818-y

**Published:** 2024-03-11

**Authors:** Ronaldo Silva Gomes, Ronaldo Machado Júnior, Cleverson Freitas de Almeida, Rebeca Lourenço de Oliveira, Moysés Nascimento, Maicon Nardino, Wellington Ferreira do Nascimento, Derly José Henriques da Silva

**Affiliations:** 1 Agronomy Department, Federal University of Viçosa-UFV, PH Rolfs Avenue, Viçosa, MG 36570-000 Brazil; 2 Statistics Departament, Federal University of Viçosa-UFV, PH Rolfs Avenue, Viçosa, MG 36570-000 Brazil; 3 Science Center of Chapadinha, Federal University of Maranhão- UFMA, 222 Rode, km 04, Chapadinha, MA 65500-00 Brazil

**Keywords:** Computational intelligence, *Cucurbita*, Fatty acid profile, Germplasm collection, Kohonen maps, Plant sciences, Plant breeding

## Abstract

With widespread cultivation, *Cucurbita moschata* stands out for the carotenoid content of its fruits such as *β* and *α*-carotene, components with pronounced provitamin A function and antioxidant activity. *C. moschata* seed oil has a high monounsaturated fatty acid content and vitamin E, constituting a lipid source of high chemical–nutritional quality. The present study evaluates the agronomic and chemical–nutritional aspects of 91 accessions of *C. moschata* kept at the BGH-UFV and propose the establishment of a core collection based on multivariate approaches and on the implementation of Artificial Neural Networks (ANNs). ANNs was more efficient in identifying similarity patterns and in organizing the distance between the genotypes in the groups. The averages and variances of traits in the CC formed using a 15% sampling of accessions, were closer to those of the complete collection, particularly for accumulated degree days for flowering, the mass of seeds per fruit, and seed and oil productivity. Establishing the 15% CC, based on the broad characterization of this germplasm, will be crucial to optimize the evaluation and use of promising accessions from this collection in *C. moschata* breeding programs, especially for traits of high chemical–nutritional importance such as the carotenoid content and the fatty acid profile.

## Introduction

Winter squash (*Cucurbita moschata* D.) is one of the most socioeconomically and nutritionally important vegetables in the *Cucurbita* genus, cultivated worldwide. It stands out for the nutritional value of its fruits, characterized by a pronounced content of bioactive components such as *β* and *α*-carotene^1,2^. These precursors have the highest provitamin A activity^3^ and show high antioxidant activity^4^. Considered one of the main sources of *β* and *α*-carotene among the vegetables consumed in Brazil^5^, *C. moschata* is one of the species prioritized in programs aimed at vitamin A biofortification and circumventing vitamin A deficiencies^6^.

Studies also point to the potential of using *C. moschata* seeds in human food production, emphasizing their high levels of unsaturated fatty acids (UFA) and bioactive components. The seed oil of *C. moschata* consists of approximately 70% UFA and a high content of monounsaturated fatty acid (MUFA) such as oleic acid^7,8^. Such characteristics make this oil an excellent option in human nutrition, particularly in replacing lipid sources harmful to health, such as those with a predominance of saturated fatty acids. It has also been demonstrated that *C. moschata* seed oil contains a high content of vitamin E components, such as *α-* and *γ*-tocopherol, and carotenoids; which are bioactive components widely known for their beneficial effects on human health^9,10^.

*C. moschata,* a cosmopolitan vegetable crop, is cultivated in a wide geographic range, from tropical to temperate regions^11^. Archaeological evidence points to the presence and food use of this vegetable in Latin America, specifically Colombia and Ecuador, for more than 7000 years^12,13^. Its cultivation spread throughout Latin America, mostly to countries such as Argentina, Peru, and Brazil^11,14^. In Brazil, *C. moschata* cultivation is widespread, covering different edaphoclimatic conditions and production systems^15^, mainly family-based production. Linked to this, studies have highlighted the remarkable variability in the agronomic characteristics, resistance against phytopathogens, and chemical–nutritional traits of fruits and seeds of the *C. moschata* germplasm found in Brazil^16–18^.

The Vegetable Germplasm Bank of the Federal University of Viçosa (BGH-UFV) was founded in 1966. Since then, it has carried out germplasm collection for a period of more than five decades, covering different geographic regions of Brazil^19^. Currently, this bank maintains a collection of around 350 accessions of *C. moschata*, which represents a substantial sample of the Brazilian germplasm and is one of the largest collections of this species in the country^19,20^.

The usefulness of plant germplasms conserved in banks depends on the quantity and quality of information associated with it, corroborating the efforts aimed at its proper evaluation. However, common restrictions in the evaluation of germplasms kept in banks, such as financial limitations and the lack of human resources, generally limit this evaluation^21^. The application of computational intelligence, and more specifically of artificial neural networks (ANNs), is a promising tool for evaluating and managing plant germplasms conserved in banks^22,23^. This tool has aroused interest because it can map non-linear systems, extracting the particularities of these systems from information such as measurements, samples, or patterns. Interest in applying ANNs also stems from their ability to adapt through experience, learning ability, generalization ability, and fault tolerance. The fact that their implementation is not linked to the experimentation process and the nature of the data set, allowing them to circumvent limitations often associated with multivariate analyzes, is another advantage in using ANNs^24^.

The emphasis placed on the conservation of plant germplasm in banks has resulted in the establishment of extensive collections. On the other hand, it has been highlighted that the use optimization of a collection is inversely related to its size^25^. Therefore, with a view to improving the use, accessibility, and conservation of accessions maintained in germplasm banks, Frankel^26^ proposed the concept of the core collection. Brown et al.^27^ Defined the term core collection as a set of accessions chosen from a germplasm collection to represent the maximum genetic variability of the initial collection, with minimum redundancy. The establishment of core collections consists of a procedure widely used in collections of plant germplasm, covering different species and based on different methodologies^28–30^. *Cucurbita moschata* crop is characterized by vigorous growth, demanding large areas and intense labor for the agro-morphological evaluation of its germplasm. With this, the implementation of ANNs represents a promising approach for optimizing the evaluation and management of this vegetable germplasm.

Given the above, this present study evaluated the agronomic and chemical–nutritional aspects of 91 accessions of *C. moschata* maintained at the BGH-UFV. The study analyzed the variability of the germplasm, and established a core collection based on multivariate approaches and the implementation of ANNs, aiming at optimizing the use and management of this germplasm.

## Materials and methods

### Origin of germplasm and conduct of the experiment

This work initially comprised the agro-morphological assessment of part of the *C. moschata* collection maintained at the BGH-UFV, including 91 accessions from different regions of Brazil, mostly landraces collected from family-based properties (Table 1). This germplasm was previously collected from several collections, as detailed by (Silva 2001)^19^.

**Table 1:** Origin of part of the *C. moschata* accessions kept in the Vegetable Germplasm Bank of the Federal University of Viçosa.

Regions	Accesseions and states of origin
South	BGH-7219A(PR), BGH-7668(PR), BGH-1461A(SC), BGH-6749(SC)
Southeast	BGH-5472A(SP), BGH-5541(SP), BGH-5556A(SP), BGH-5548A(SP), BGH-5453A(SP), BGH-5473A(SP), BGH-5544A(SP), BGH-5591A(SP), BGH-5593(SP), BGH-5596A(SP), BGH-5440A(SP), BGH-5485A(SP), BGH-5455A(SP), BGH-5598A(SP), BGH-5493A(SP), BGH-5494A(SP), BGH-5559A(SP), BGH-5499A(SP), BGH-5530A(SP), BGH-5606A(SP), BGH-5442(SP), BGH-5538(SP), BGH-5554A(SP), BGH-5301(SP), BGH-5451(SP), BGH-5528(SP), BGH-5551(SP), BGH-5552(SP), BGH-5553(SP), BGH-5560A(SP), BGH-5597(SP), BGH-900(SP), BGH-5497(SP), BGH-5603(SP), BGH-5466(SP), BGH-5456A(SP), BGH-4459A(MG), BGH-4281(MG), BGH-4454A(MG), BGH-6116(MG), BGH-4590A(MG), BGH-1927(MG), BGH-4681A(MG), BGH-4610A(MG), BGH-5361A(MG), BGH-5247A(MG), BGH-6115(MG), BGH-1004(MG), BGH-4516(MG), BGH-5248(MG), BGH-5648(MG), BGH-5659A(MG), BGH-4453(MG), BGH-4607A(MG), BGH-6155(MG), BGH-4287A(MG), BGH-4598A(MG), BGH-5224A(MG), BGH-6117A(MG), BGH-305A(MG), BGH-3333A(RJ), BGH-291(RJ), BGH-5051(RJ), BGH-1961(ES), BGH-1945A(ES), BGH-1992(ES)
Midwest	BGH-5616A(DF), BGH-5630A(DF), BGH-5624A(DF), BGH-315(DF), BGH-5638(DF), GBH-5694(DF), BGH-5639(DF), BGH-6590(GO), BGH-6587A(GO), BGH-6595(GO), BGH-6593(GO), BGH-6794(GO), BGH-6594(GO)
North East	BGH-6099(RN), BGH-6096(RN), BGH-5653(BA), BGH-117(BA), BGH-1749(BA), BGH-95(BA), BGH-5649A(BA), BGH-5240(BA)
Brazil	Jabras* (BR), Tetsukabuto* (BR), Jacarezinho* (BR), Maranhão* (BR)

The two letters associated with the accessions names refer to Brazilian states where the accessions were collected, namely Paraná (PR), Santa Catarina (SC), São Paulo (SP), Minas Gerais (MG), Rio de Janeiro (RJ), Espírito Santo (ES), Distrito Federal (DF), Goiás (GO), Rio Grande do Norte (RN), and Bahia (BA). *These genotypes are commercial cultivars widely cultivated in Brazil.

### Agro-morphological evaluations

The agro-morphological evaluation was carried out in a field experiment conducted from January to July 2016 at the Experimental Unit of the Department of Agronomy at UFV-“Horta Velha” (20° 4524″ S, 42° 5045″ W; altitude, 648.74 m). The soil in the experimental area is classified as dystrophic Red Yellow Oxisol with a flat topography, and the climate in the region is Cwb, with an average annual temperature of 19.4 °C and annual precipitation of approximately 1200 mm.

The evaluation of the genotypes comprised vegetative traits, production, and chemical–nutritional aspects of fruits, seeds, and seed oil. Details about the agro-morphological descriptors used in the evaluation of germplasm are provided in Supplementary Table 2. The genotypes were also evaluated for multi-categorical traits and details about these traits are provided in Supplementary Table 3. The accessions were evaluated together with four commercial cultivars used as controls: the hybrids Tetsukabuto and Jabras (*C. moschata* × *C. maxima*) and the cultivars Jacarezinho and Maranhão. These genotypes were evaluated using Federer's augmented block design^31^, with five replications for each control. The four controls were randomly distributed in each block, and the accessions were randomly distributed among all the blocks in equal numbers. The experiment was established using a spacing of 3 × 3 m between plants and rows, with five plants per plot. The production and transplanting of seedlings and the cultural treatments were carried out in accordance with the local recommendations for the crop^32^.

The agro-morphological evaluations were carried out on the three central plants of each plot, using three fruits per plant. The carotenoid content was estimated based on the analysis of colorimetric parameters of fruit pulp, using a manual tristimulus colorimeter (Color Reader CR-10; Konica Minolta, Tokyo, Japan). This assessment was performed as detailed by^18^, according to the equations proposed by^33^, described below:

$$C=\sqrt{{a}^{2}+{b}^{2}}$$

$$TC = {6},{1226} + {1},{71}0{6}^*{\text{a}}$$

$$L = - {6}.{3743} + 0.{2818}^*{\text{C}}$$

where *C* corresponds to the saturation or chroma of fruit pulp; *a* and *b* correspond to the contribution of red and yellow to the color of fruit pulp (dimensionless), respectively; *TC* corresponds to the total content of carotenoids, and *L* corresponds to the lutein content of fruit pulp, both expressed in μg g^−1^ of fresh fruit mass.

The seed oil content (SOC) was determined using an extractor (ANKOM XT15, ANKON, Macedon, United States), according to a standard method from the Association of Official Analytical Chemists (AOAC), described by^34^. The extraction of seed oil was carried out using mechanical pressing, according to the methodology by^18^, and the fatty acid profile was analyzed using gas chromatography (GC). GC was performed using the GC-17A gas chromatograph (Shimadzu Corporation, Kyoto, Japan), equipped with an automatic insertion platform, flame ionization detector, and a Carbowax capillary column (30 m × 0.25 nm). Chromatography was performed under injection and detection temperatures of 230 and 250 °C, respectively. Column operation started at 200 °C, with an increase of 3 °C·min^−1^, until reaching a temperature of 225 °C. Nitrogen was used as a carrier gas with a flow rate of 1.3 L·min^−1^, and the concentration of each methyl ester was determined as a percentage of the relative peak area.

### Implementation of restricted maximum likelihood procedures and the best linear unbiased prediction for analysis of agromophological data

Agromophological data were analysed from restricted maximum likelihood (REML) procedures and the best linear unbiased prediction (BLUP). This analysis was carried out using the R program and the package lme4^35^. The genotypic values of accessions (BLUP) and controls (BLUES) were obtained from the BLUP, while the estimates of variance components were obtained from the REML, based on the following model:

$$y = {\text{W}}b + {\text{X}}a + {\text{Z}}t + e$$

where, *y* representes the phenotypic data vector, *b* representes the vector of blocks effect ssumed to be random, *a* representes the vector of accessions effect assumed to be random, *t* representes the vector of controls effect assumed to be fixed, and *e* represents the error vector. The letters W, X and Z represents the incidence matrices of parameters *b*, *a*, and *t*, respectively, with the data vector *y***.** Both multivariate and ANNs analysis were carried out using the estimates of BLUP and BLUES.

The estimates of variance components comprised only the genotypic variance (*σ*^2^_*g*_). Heritability was obtained based on the following estimator: *h*^2^ = 1 − (*Pev*/*σ*^2^_*g*_), where *Pev* represents the prediction of error variance^36^.

### Analysis of genetic variability using multivariate approaches and artificial neural networks

Multivariate analysis included the grouping of genotypes and the distribution of accessions in relation to principal components. Multivariate analysis of variability was carried out using both quantitative and multi-categorical information. For quantitative data, the distance matrix between genotypes was obtained using the standardized average Euclidean distance, from the estimates of BLUPs and BLUES. For multi-categorical data, the distance matrix was obtained from the arithmetic complement of the simple coincidence index. These matrices were then summed, resulting in a single distance matrix. For the sum of the matrices, they were standardized and each one received an equal weight in the summation procedure. The choice of the grouping method was based on cophenetic correlation, opting for the grouping that provided highest cophenetic correlation coefficient; while the determination of number of groups to be formed in clustering was based on the methodology proposed by^37^. Multivariate analysis were performed with the help of Matlab^38^ and the Genes software^39^.

A principal component analysis was implemented in order to identify the distribution of accessions in relation to the principal components. This analysis considered the data of quantitative and multi-categorical traits, according to the methodology of^40^; and was implemented with the help of Matlab^38^.

The analysis of the genetic variability organization through neural networks was carried out using Kohonen self-organizing maps (SOM). For this, different two-dimensional hexagonal topological maps were tested in which the N units (neurons) were allocated considering the number of rows and columns, ranging from 1 to 7. This procedure was based on the understanding that defining the topological map and, consequently, the number of neurons and parameters should be based on the researcher's experience, and trial and error methods^41^. Next, the selection of the best network architecture from 2000.00 training sessions for each of the combinations was carried out. The defined network topology had a hexagonal neighborhood. Network analysis was performed with the help of Matlab^38^ and the Genes software^39^.

Core collections were established from the random sampling of accessions from the full collection using sampling intensities of 10, 15, 20, and 25%. Thus, 9, 14, 18, and 23 accessions were sampled from the full collection to form the core collections with sampling intensities of 10, 15, 20, and 25%, respectively. The sampling of accessions for the establishment of the core collection was random and with no replacement. The validation of core collections was carried out from the comparison with the complete collection, based on the parameters obtained for the agro-morphological characteristics such as mean and variance^29^. Means and phenotypic variances of variables in the complete collection and nuclear collections were estimated with the aid of the Genes software^39^.

### Collection and use of any plant materials statement

The authors declare that the plant collection and use was carried in accordance with all the relevant guidelines.

## Results

### Phenotypic range and heritability of traits

**Figure 1:**
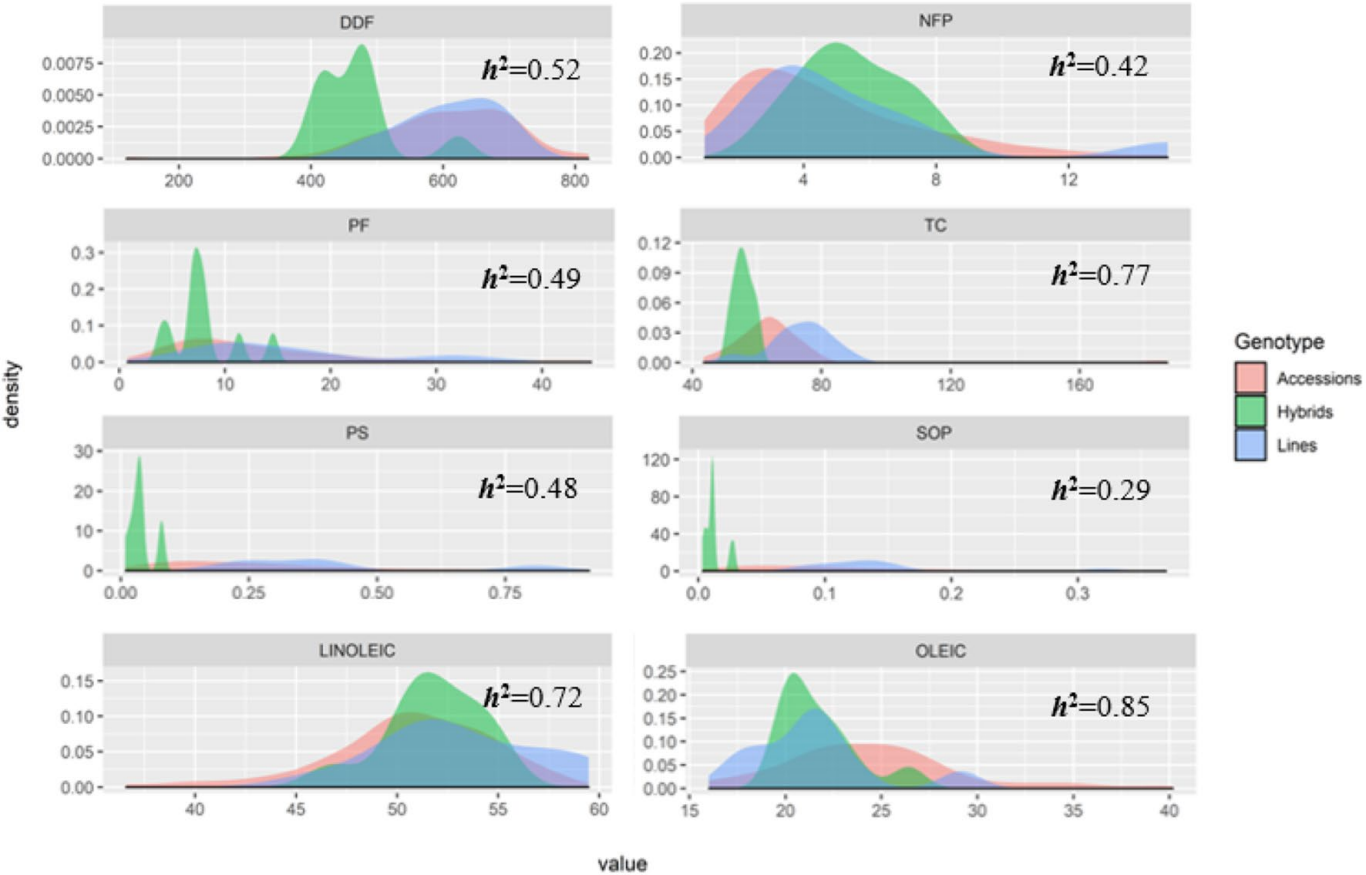
Frequency distribution of characteristics associated with fruit production and chemical–nutritional aspects of fruit pulp and seed oil. DDF, Accumulated degree days for flowering; NFP, Number of fruits per plant; PF, Productivity of fruits; TC, Total carotenoid content of fruit pulp; PS, Productivity of seeds; SOP, Seed oil productivity; LAC, Linoleic acid content, and OAC, oleic acid content.

Based on the distribution analysis of traits, we observed a high phenotypic range for fruit production traits and chemical–nutritional aspects of fruit pulp and seed oil (Fig. 1). These amplitudes were especially higher for the productivity of fruits (PF), the total carotenoid content of fruit pulp (TC), and the oleic and linoleic fatty acid contents. Associated to this, these traits expressed significant genotypic variances and heritability estimates ranging from high to very high (Fig. 1).

Most of the traits expressed greater amplitude between the accessions compared with the hybrids or lines used as controls.

### Clustering of genotypes and principal components analysis from multivariate approach

**Figure 2:**
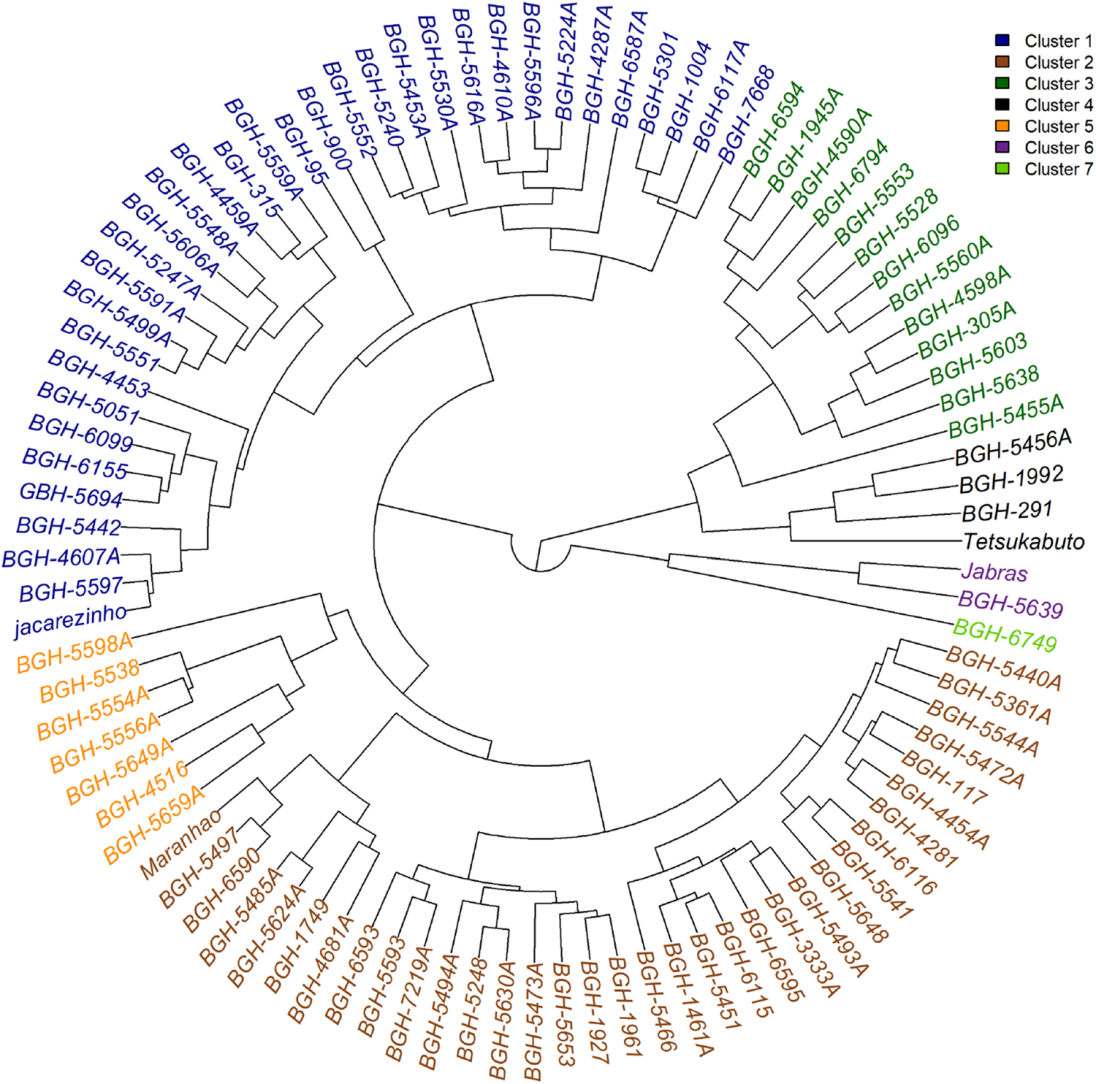
Grouping of accessions and controls based on a multivariate approach.

The unweighted pair-group method using arithmetic averages (UPGMA) grouping method provided one of the highest cophenetic correlation indexes (> 0.7) and was adopted for the grouping of genotypes. Analysis of the variability using the multivariate approach showed that accessions and controls were grouped into seven groups. Groups 1 and 2 were the largest groups consisting of 33 and 37 genotypes, respectively (Fig. 2). Group 7 contained only the BGH-6749 genotype and was the smallest group.

Group 7 had the lowest number of accumulated degree days for flowering (DDF), followed by groups 6 and 4. These groups also had the lowest averages for DDF. Group 5 contained the genotype with the highest productivity of fruits (44.67 t. ha^−1^) and was the group with the highest average productivity of fruits (21.74 t. ha^−1^).

Groups 2 and 3 contained the genotypes with the highest average for total carotenoid content in fruit pulp, with contents of 187.21 and 181.17 μg g^−1^ of fresh mass, respectively. Group 4 contained the genotype with the highest content of oleic fatty acid in the oil (40.18%) and was also the group with the highest average for this characteristic (26.28%). Group 4 also contained the genotype with the lowest linoleic fatty acid content.

**Figure 3:**
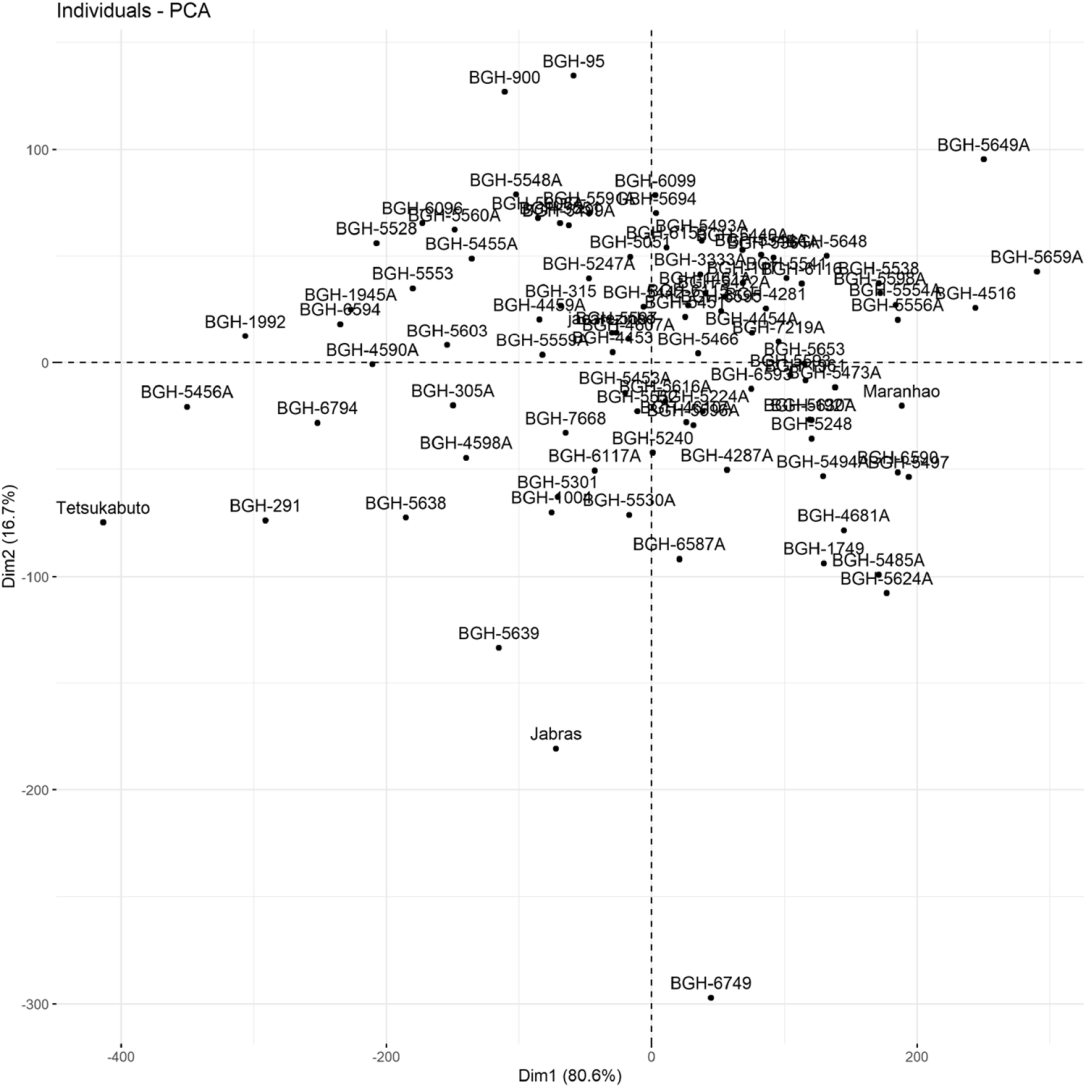
Genetic variability of the 91 accessions of *C. moschata* kept in BGH-UFV from principal components (multivariate approach), showing the dispersion of genotypes in relation to the first two principal components.

Figure 3 demonstrates the distribution of accessions in relation to the first two principal components (PC), emphasizing the analysis of genotype variability based on the multivariate approach. PC analysis highlighted accessions BGH-5456A, BGH-1992, and BGH-291 as those with the highest loads in the first PC. The first PC explained 80.6% of the total variation of genotypes in relation to agro-morphological characteristics, and the second PC explained 16.7%.

### Organization of the accession’s variability from ANNs

**Figure 4:**
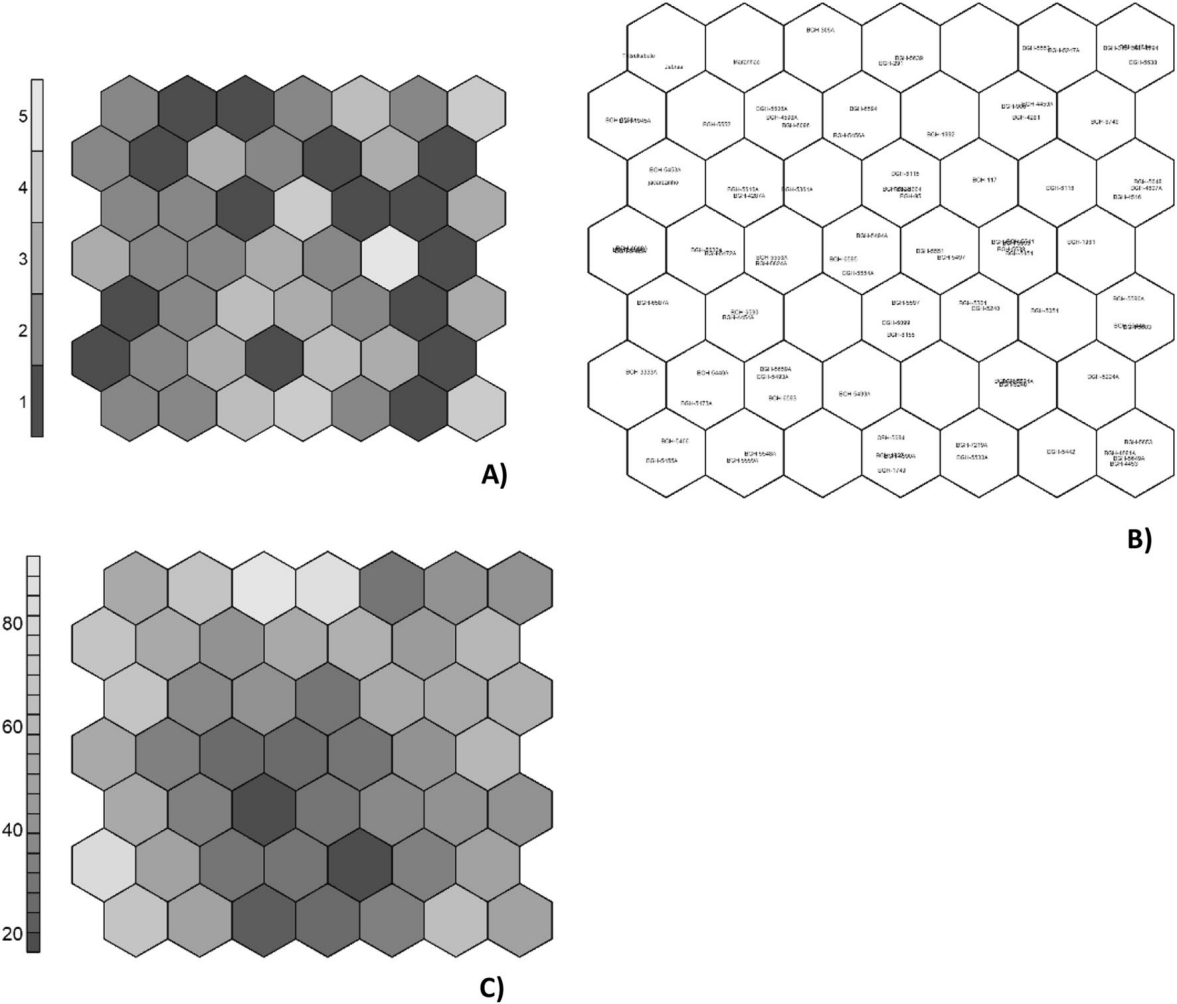
Kohonen’s self-organizing map demonstrating the concentration and genetic distances of genotypes in neurons. Distribution of genotypes in neurons (**A**,**B**) and genetic distance between the genotypes of each neuron (**C**). In Fig. 4A, the lighter color denotes greater number of accessions per neuron, while the darker color denotes smaller number of accessions per neuron. The lighter color denotes a greater distance between the genotypes in the neuron, while the darker color denotes a smaller distance in Fig. 4C.

Figure 4 shows the variability of accessions from ANN and SOM. It is observed that each neuron concentrated a similar number of accessions and controls, demonstrating an equitable concentration of these genotypes in the neurons (Fig. 4A,B, Table 2). ANNs analysis provided information about the genetic distance between the accessions and controls in each neuron. A tendency for genotypes with greater genetic distance to concentrate in the extreme neurons was observed (Fig. 4C).

**Table 2:** Concentration of genotypes in neurons from Kohonen’s self-organizing map, as shown in Fig. 4A,B.

Neurons	Genotypes	Neurons	Genotypes
1	BGH-5466, BGH-5455A	26	BGH-5551, BGH-5606
2	BGH-5548A, BGH-5630A	27	BGH-1749, BGH-4681, BGH-624A
3	–	28	BGH-1961, BGH-1927
4	BGH-5694, BGH-4590A, BGH-5591A	29	BGH-5453, Jacarezinho
5	BGH-7219, BGH-5554	30	BGH-5494A, BGH-4287A
6	BGH-5442	31	BGH-5361
7	BGH-1461A, BGH-5648, BGH-5649A, BGH-4453	32	BGH-5528, BGH-6115, BGH-6595, BGH-95, BGH-6116
8	BGH-3333A	33	BGH-117
9	BGH-6593, BGH-5473A	34	BGH-6749
10	BGH-5659A, BGH-5485	35	BGH-5593, BGH-4607A, BGH-516
11	BGH-6099	36	BGH-1945A, BGH-1004
12	–	37	BGH-5552
13	BGH-5653, BGH-5440A, BGH-5544A	38	BGH-5472, BGH-5598, BGH-5603
14	BGH-5224A	39	BGH-6594, BGH-5456A
15	BGH-6587A	40	BGH-1992
16	BGH-6590, BGH-5497	41	BGH-900, BGH-4454A, BGH-4281
17	–	42	BGH-5248
18	BGH-5451, BGH-5493A, BGH-6155	43	Tetsukabuto, Jabras
19	BGH-5301, BGH-5240	44	Maranhão
20	BGH-5051	45	BGH-305A
21	BGH-5530, BGH-5639, BGH-5560A	46	BGH-5638, BGH-291
22	BGH-5616A, BGH-5596A, BGH-4610A, BGH-6117A	47	–
23	BGH-5499A, BGH-6096	48	BGH-315, BGH-5553, BGH-5247A, BGH-5597
24	BGH-5559A, BGH-4459A	49	BGH-4598A, BGH-6794, BGH-668
25	BGH-5541, BGH-5538, BGH-5556A		

### Establishment and validation of the core collection

Table 3 shows the list of accessions of each core collection obtained from the different sampling intensities. Validation of the core collections was performed by comparing the mean and variance of the complete collection and the mean and variance of each core collection.

**Table 3:** List of accessions in core collections formed from different sampling intensities.

Core collection (%)	Accessions
10	BGH-1992, BGH-4610A, BGH-5556A, BGH-4454A, BGH-5639, BGH-5455A, BGH-315, BGH-5538, BGH-6794
15	BGH-4287A, BGH-4598A, BGH-5548A, BGH-1461A, BGH-5473A, BGH-5630A, BGH-4590A, BGH-5639, BGH-5240, BGH-5552, BGH-5499A, BGH-5456A, BGH-5598A, BGH-5442
20	BGH-5051, BGH-5472A, BGH-4454A, BGH-3333A, BGH-5361A, BGH-5630A, BGH-4453, BGH-5248, BGH-5552, BGH-5473A, BGH-5497, BGH-5548A, BGH-5544A, BGH-5541, BGH-7668, BGH-900, BGH-5596A, GBH-5694
25	BGH-6590, BGH-4598A, BGH-5596A, BGH-6117A, BGH-5361A, BGH-6096, BGH-4454A, BGH-4281, BGH-6587A, BGH-5456A, BGH-5554A, BGH-5598A, BGH-5530A, BGH-5638, BGH-315, BGH-6155, BGH-6794, BGH-4607A, BGH-5552, BGH-4610A, BGH-5556A, BGH-4287A, BGH-6116

In general, the core collection obtained under a sampling intensity of 15% (15% CC) presented a mean and variance closer to those of the complete collection. The means and variances for degree days accumulated for flowering (DDF), number of fruits per plant (NFP), mass of seeds per fruit (MSF), productivity of seeds (PS), and SOP characteristics using 15% CC were very close to those of the complete collection (Table 4).

**Table 4:** Means and variances of agro-morphological traits in the different core collections.

Traits	Complete collection		Core collections							
			10%		15%		20%		25%	
	Means	Variances	Means	Variances	Means	Variances	Means	Variances	Means	Variances
DDF	606.321	10,940.52	583.50	12,987.21	575.99	10,352.13	640.50	5121.60	590.81	8080.36
NFP	4.78	8.35	6.31	15.10	5.54	6.12	5.74	6.43	5.79	10.05
PF	12.76	72.84	11.78	27.15	11.52	34.12	17.66	103.18	12.12	32.92
TC	65.76	382.89	79.62	1676.02	72.52	1029.72	61.54	71.60	69.16	662.89
MSF	51.67	468.73	46.24	853.20	49.93	414.66	56.09	423.67	50.65	386.98
PS	0.27	0.040	0.36	0.09	0.29	0.03	0.36	0.05	0.33	0.06
SOP	0.10	0.00	0.14	0.02	0.11	0.00	0.14	0.01	0.13	0.01
LAC	50.65	16.70	51.39	16.54	50.40	24.88	49.05	23.56	50.03	24.82
OAC	24.54	21.54	23.51	19.71	25.10	28.85	26.68	32.67	25.18	31.47

DDF, Accumulated degree days for flowering; NFP, Number of fruits per plant; PF, productivity of fruits; TC, Total carotenoid content of fruit pulp; MSF, Mass of seeds per fruit; PS, Productivity of seeds; SOP, Seed oil productivity; LAC, Linoleic acid content; and OAC, oleic acid content.

## Discussion

The high phenotypic ranges observed in this study for traits such as fruit productivity, total carotenoid content of fruit pulp, and oleic and linoleic fatty acid levels are in line with the genetic variability observed in previous studies of the *C. moschata* germplasm^16–18,42^.

Accessions BGH-5455A and BGH-5598A expressed the highest carotenoid contents with 187.21 and 181.17 μg g^−1^ of fresh pulp mass, respectively. This result is much higher than those reported in previous studies^2,43^. For example, the study involving the characterization of 55 accessions of *C. moschata*, also maintained by the BGH-UFV, reported a total content of carotenoids in the fruit pulp not exceeding 118.70 μg g^−1^ of fresh pulp mass^44^. On the other hand, when evaluating the *C. moschata* germplasm from Northeastern Brazil, Carvalho et al.^1^ reported averages of up to 404.98 μg g^−1^. The differences observed in the total content of carotenoids in fruit pulp between the present and previous studies may be mainly associated with the genetic aspects of the germplasm evaluated in each study. Studies with *C. moschata* generally reported high levels of carotenoids in fruit pulp^1,45^, particularly *β*- and *α*-carotene. These components are known for their important biological functions, such as provitamin A^46^ and antioxidant activity^4^.

Accessions BGH-5456A, BGH-3333A, BGH-5361A, and BGH-5472A expressed the highest levels of oleic acid. The emphasis on the analysis of the fatty acid profile of *C. moschata* aims at exploring the potential of this vegetable as an oleaginous crop. Consisting of approximately 75% of UFA and with a high content of MUFA such as oleic acid^7,8^, the oil from *C. moschata* seeds is an excellent substitute for lipid sources with high levels of saturated fatty acids, harmful to human health. Corroborating this, studies demonstrate the association between the consumption of lipid sources composed predominantly of saturated fatty acids and the high risk of cardiometabolic pathologies, particularly cardiovascular diseases and type II diabetes mellitus^47,48^. This has encouraged the replacement of saturated lipids in human food with UFA, with a particular focus on vegetable oils—the main source of UFA in the human diet.

Using multivariate analyzes and ANNs highlighted the high variability of *C. moschata* accessions. Clustering using a radial dendrogram allowed the identification of groups with the most promising averages in terms of accumulated DDF, PF, TC, and fatty acid profile (Fig. 2). The analysis of variability using PC corroborated the accession grouping pattern using the dendrogram, highlighting accessions BGH-5456A, BGH-1992, and BGH-291 as the most divergent (Fig. 3).

The analysis of the organization of the accession’s variability from ANNs corroborated the variability observed from the multivariate approach. This was confirmed by the concentration of a similar number of accessions along the neurons (Fig. 4A,B). This demonstrates that the adopted network architecture, consisting of seven columns and seven rows, efficiently organized the variability of the genotypes. Similar to the present study, a series of studies with Kohonen SOM also defined their topology randomly or by trial and error^22,49,50^. With this, it is assumed that the method to find the best architecture should be established judiciously. This is because different results can be obtained each time a SOM is used, given that networks have random synaptic weights at the beginning of training^22^.

Analysis using ANNs identified a tendency for genotypes with greater genetic distance to concentrate in the most extreme neurons (Fig. 4C), information that will support the establishment and validation of the core collections. Thus, genotypes concentrated in the extreme neurons express greater genetic distance. ANNs analysis enabled the organization of the genotypes into closer groups than those obtained from the radial dendrogram grouping (Fig. 2), proving to be more efficient in identifying similarity patterns and in organizing the proximity of genotypes between groups. Close to this, Santos et al.^22^ also used the SOM technique as an alternative method to assess genetic diversity in rice breeding programs. However, it should be noted that there is the possibility of greater variation in the allocation of genotypes in neurons as the number of neurons increase ^51^.

The variability observed among the genotypes of *C. moschata* in the present study is in line with previous studies with this species, characterized by high genetic variability, reflected, at first, in the variation of morphological aspects of plants and fruits. Studies have highlighted the variability of the Brazilian germplasm of *C. moschata*^16–18^, possibly a result of the adaptation of this germplasm to a wide ecological range found in the country, consisting of different edaphoclimatic conditions^15^. In addition, the occurrence of natural hybridization between populations also contributes to the variability in the germplasm of this vegetable^18^.

When establishing core collections, they must be evaluated regarding their ability to maintain the existing variability in the complete collection^29,30^. The averages and variances of agro-morphological characteristics of 15% CC were closest to the averages and variances of the complete collection, particularly in relation to DDF, NFP, MSF, PS, and SOP (Table 4). The 15% CC variances tended to be higher than the complete collection variances for most traits, which indicates that with this sampling intensity, the core collection effectively preserved the complete collection's genetic variability.

The validation of nuclear collections can be carried out using different approaches, such as the analysis of the amplitude coincidence index^30,52^, and are based on parameters analysis such as mean, variance, and amplitude^29,53,54^. For example, when proposing the establishment of a core collection based on the US Department of Agriculture soybean germplasm collection, Oliveira et al.^29^ emphasized the analysis of mean, variance, and amplitude observed in core collections as an approach for their validation. In this sense, Frankel^55^ highlighted that the sampling strategy is efficient when the core collection retains at least 80% of the original amplitude for a trait.

The establishment of a core collection aims to maintain the greatest possible variability from a minimum number of accessions, thus providing greater efficiency in identifying useful genetic diversity by breeders and other scientists. Given this, it is assumed that the 15% CC was effective since it presented means and variances very close to those of the complete collection and a number of accessions considerably lower than the full collection^29,56^.

According to^27^, establishing a core collection provides advantages for both collection curators and breeders. With the proposal of a core collection, two hierarchical levels are established, namely the core collection and the complete collection. From this, the curators can prioritize conservation activities such as germination and regeneration tests, in the core collections, in addition to concentrating efforts in the characterization and evaluation of the accessions of these collections. For breeders, evaluations of core collections often become less onerous due to the smaller number of accessions in these collections.

With the present study, the agro-morphological characterization of the collection of *C. moschata* maintained at the BGH-UFV approaches its conclusion^18,44,57^. Constituting a substantial sample of the Brazilian germplasm of *C. moschata* and one of the largest collections of this species in the country^20^, the characterization of this collection has covered the evaluation of an extensive set of characteristics, including the analysis of resistance against important phytopathogens of the crop, fruit and seed productivity; as well as chemical–nutritional aspects of fruits, seeds and seed oil^18,44,57^. Previous studies with the collection of *C. moschata* at the BGH-UFV allowed the identification of promising accessions as sources of genes for genetic improvement of this species.

The implementation of ANNs in the present study proved to be a useful tool to base the establishment of core collections, allowing a clearer distinction of the formed groups compared to the multivariate approach. Implementing ANNs for analyzing the organization of germplasm variability initially brings the advantage of mapping even trends or performances that do not follow linear behaviors^58^. Additionally, multivariate approaches bring disadvantages such as their association with the experimentation process and the nature of the data set. Therefore, a series of factors related to how the experimentation is conducted can compromise the efficiency of these analyses. For example, different genetic distance indices might be recommended for analyzing the diversity of a set of genotypes, depending on the statistical design in which they were evaluated. The Euclidean distance index, for example, is indicated for cases in which samples under evaluation have not been evaluated with repetition^59^, and in this case, the multivariate analysis does not include environmental errors that possibly have influenced the average results of samples. On the other hand, if there was repetition, the Mahalanobis distance is recommended^59^, which allows environmental errors to be contemplated in the multivariate analysis. The use of distance measures, such as the Euclidean ones, is restricted to quantitative data and recommended for cases in which there is no correlation between the variables, that is, for cases in which the variables are independent.

*C. moschata* crop presents characteristics that make the evaluation of its germplasm challenging. This species is characterized by branches with vigorous growth and long internodes^32,60^, which requires an extensive area for the evaluation of a reduced number of accessions, making the process costly. On the other hand, as already explained, the fruits and seeds of *C. moschata* express high nutritional value. Its fruits are characterized by a high content of carotenoids such as *β*- and *α*-carotene^1,61^, components with high provitamin A and antioxidant function^4,46^. Moreover, the seed oil of *C. moschata* consists of approximately 75% of UFA and has a high content of MUFA such as oleic acid^7,8^, components that are beneficial to human health. The establishment of the core collection proposed in the present study will be crucial to optimize the evaluation and use of promising accessions from this collection, especially for characteristics of high chemical–nutritional importance, such as the carotenoid profile of fruit pulp and the fatty acid profile of seed oil. The core collection could also be used as a source of alleles for genetic improvement programs of *C. moschata* and other cucurbits.

## Conclusion

The accessions of *C. moschata* expressed a considerable phenotypic range for productivity of fruits, total carotenoid content of fruit pulp, and oleic and linoleic fatty acid contents, which enabled the identification of promising accessions for use as a source of genes for genetic improvement of these traits.

Multivariate analyzes and the approach using ANNs highlighted the high variability of *C. moschata* accessions evaluated in this study. The variability organization of accessions from ANNs corroborated the variability of accessions observed from the multivariate approach. This demonstrates that the network architecture adopted efficiently organized the genotype variability. ANNs were able to organize the genotypes into closer groups than those obtained from the radial dendrogram grouping, proving to be more efficient in identifying similarity patterns and in organizing the proximity of genotypes between groups. This information was fundamental to supporting the core collections' establishment and validation.

The averages and variances of agro-morphological traits using 15% CC were those closest to the averages and variances of the complete collection, particularly in relation to DDF, NFP, MSF, PS, and SOP, demonstrating that this core collection was efficient in maintaining the variability of accessions. Establishing the 15% CC will be crucial to optimize the evaluation and use of promising accessions from this collection, especially for traits of high chemical–nutritional importance, such as the carotenoid profile of fruit pulp and the fatty acid profile of seed oil.

### Supplementary Information

Supplementary Information.

Supplementary Tables.

## Data Availability

The authors declares that all data generated or analysed during this study are included in this published article [and its Supplementary information files].

## References

[1] Carvalho LMJ (2012). Total carotenoid content, *α*-carotene and *β*-carotene, of landrace pumpkins (*Cucurbita moschata* Duch): A preliminary study. Food Res. Int..

[2] Azevedo-Meleiro CH, Rodriguez-Amaya DB (2007). Qualitative and quantitative differences in carotenoid composition among *Cucurbita moschata*, *Cucurbita maxima*, and *Cucurbita pepo*. J. Agr. Food Chem..

[3] Khillan JS (2014). Vitamin A/retinol and maintenance of pluripotency of stem cells. Nutrients.

[4] Jayedi A, Rashidy-Pour A, Parohan M, Zargar MS, Shab-Bidar S (2018). Dietary antioxidants, circulating antioxidant concentrations, total antioxidant capacity, and risk of all-cause mortality: A systematic review and dose-response meta-analysis of prospective observational studies. Adv. Nutr..

[5] Rodriguez-amaya DB, Kimura M, Godoy HT, Amaya-Farfan J (2008). Updated Brazilian database on food carotenoids: Factors affecting carotenoids composition. J. Food. Compos. Anal..

[6] Saltzman A (2013). Biofortification: Progress toward a more nourishing future. Glob. Food. Secur-Agr..

[7] Jarret RL, Levy IJ, Potter TL, Cermak SC, Merrick LC (2013). Seed oil content and fatty acid composition in a genebank collection of *Cucurbita moschata* Duchesne and *C. argyrosperma* C. Huber. Plant Genet. Resour..

[8] Veronezi CM, Jorge N (2015). Chemical characterization of the lipid fractions of pumpkin seeds. Nutr. Food Sci..

[9] Veronezi C, Jorge N (2012). Bioactive compounds in lipid fractions of pumpkin (*Cucurbita* sp) seeds for use in food. J. Food Sci..

[10] Dash P, Ghosh G (2017). Proteolytic and antioxidant activity of protein fractions of seeds of *Cucurbita moschata*. Food Biosci..

[11] FAO, FAO-Food and Agriculture Organization of the United Nations https://www.fao.org/faostat/en/#data/QCL (2022). 6086142

[12] Dillehay T, Rossen J, Andres TC, Williams DE (2007). Preceramic adoption of peanut, squash, and cotton in Northern Peru. Science.

[13] Piperno DR, Stothert KE (2003). Phytolith evidence for early Holocene *Cucurbita* domestication in Southwest Ecuador. Science.

[14] IBGE-Instituto Brasileiro de Geografia e Estatística. Produção Agrícola Municipal 2017 https://sidra.ibge.gov.br/tabela/6957#resultado (2022).

[15] Gomes RS (2022). Identification of high seed oil yield and high oleic acid content in Brazilian germplasm of winter squash (*Cucurbita*
*moschata* D.). Saudi J. Biol. Sci..

[16] De Lima GKL, De Queiroz MA, Da Silveira LM (2016). Rescue of *Cucurbita* spp. germplasm in Rio Grande do Norte. Rev. Caatinga.

[17] Ferreira MG, Salvador FV, Lima MNR (2016). Parâmetros genéticos, dissimilaridade e desempenho per se em acessos de abóbora. Hortic. Bras..

[18] Gomes RS (2020). Brazilian germplasm of winter squash (*Cucurbita*
*moschata* D.) displays vast genetic variability, allowing identification of promising genotypes for agro-morphological traits. Plos One.

[19] Silva DJH, Moura MCC, Casali VWD (2001). Recursos genéticos do Banco de Germoplasma de Hortaliças da UFV: Histórico e expedições de coleta. Hortic. Bras..

[20] Fonseca MA (2015). Geographical distribution and conservation of *Cucurbita* in Brazil. Magistra.

[21] IPGRI-International Plant Genetic Resources Institute (2001). The Design and Analysis of Evaluation Trials of Genetic Resources Collections: A Guide for Gene Bank Managers.

[22] Santos IG, Carneiro VQ, Silva Junior AC, Da Cruz CD, Soares PC (2019). Self-organizing maps in the study of genetic diversity among irrigated rice genotypes. Acta Sci-Agron..

[23] Silva MJ (2020). Computational intelligence for studies on genetic diversity between genotypes of biomass sorghum. Pesqui. Agropecu. Bras..

[24] Da Silva IN, Spatti DH, Flauzino RA (2010). Redes neurais artificiais para engenharia e ciências aplicadas.

[25] Frankel OH, Soulé M (1981). Conservation and Evolution.

[26] Frankel, O. H. Genetic perspectives of germplasm conservation. In *Genetic Manipulation: Impact on Man and Society*. (ed. Arber, W. K., Llimensee, K., Peacok, W.J. & Starlinger, P.). 161–170 (Cambridge University, Cambridge, 1984).

[27] Brown, A. H. D., Spillane, C., Johnson, R. C. & Hodgkin, T. Implementing core collections principles, procedures, progress, problems and promise. In *Core collection for today and tomorrow*. (ed. Johnson, R. C. & Hodgkin, T.). 1–9 (IPGRI, Italy, 1999).

[28] Liang W, Dondini L, De Franceschi P (2015). Genetic diversity, population structure and construction of a core collection of apple cultivars from Italian germplasm. Plant Mol. Biol. Repos..

[29] Oliveira MF, Nelson RL, Geraldi IO, Cruz CD, Toledo JFF (2010). Establishing a soybean germplasm core collection. Field Crop Res..

[30] Sobreira, F. M. Divergência genética entre acessos de abóbora para estabelecimento de coleção nuclear e pré-melhoramento para óleo funcional. D. Sc. Thesis, Universidade Federal de Viçosa. 2013. Available from: https://locus.ufv.br//handle/123456789/1367

[31] Federer WT (1956). Augmented or (Hoonuiaku) designs. Hawaii. Planter’s Record..

[32] Filgueira FAR (2008). Novo manual de olericultura: agrotecnologia moderna na produção e comercialização de hortaliças.

[33] Itle RA, Kabelka EA (2009). Correlation between L* a* b* color space values and carotenoid content in pumpkins and squash (Cucurbita spp.). Hortscience.

[34] Thiex NJ, Anderson S, Gildemeister B (2003). Crude fat, hexanes extraction, in feed, cereal grain, and forage (Randall/Soxtec/Submersion Method): Collaborative study. J. AOAC Int..

[35] Bates D, Maechler M, Bolker B, Walker S (2015). Fitting linear mixed-effects models using lme4. J. Stat. Softw..

[36] Cullis BR, Smith AB, Coombes NE (2006). On the design of early generation variety trials with correlated data. J. Agric. Biol. Environ. Stat..

[37] Caliński T, Harabasz J (1974). A dendrite method for cluster analysis. Commun. Stat..

[38] MATLAB. version 7.10.0. Natick: The Math Works Inc., 2012. Software.

[39] Cruz CD (2013). Genes—A software package for analysis in experimental statistics and quantitative genetics. Acta Sci-Agron..

[40] Pagès J (2004). Analyse factorielle de données mixtes. Rev. Stat. Appl..

[41] Kohonen T (2001). Self-Organizing Maps.

[42] Hernández-Rosales HS (2020). Phylogeographic and population genetic analyses of *Cucurbita moschata* reveal divergence of two mitochondrial lineages linked to an elevational gradient. Am. J. Bot..

[43] Priori D, Valduga E, Villela JCB (2017). Characterization of bioactive compounds, antioxidant activity and minerals in landraces of pumpkin (*Cucurbita moschata*) cultivated in Southern Brazil. Food Sci. Tech-Brazil..

[44] Lima Neto, I. S. Pré-melhoramento de abobra (*Cucurbita moschata*) visando a biofortificacao em carotenoides. D. Sc. Thesis, Universidade Federal de Viçosa. 2013. Available from: https://locus.ufv.br//handle/123456789/1200

[45] Nakkanong K, Yang JH, Zhang MF (2012). Carotenoid accumulation and carotenogenic gene expression during fruit development in novel interspecific inbred squash lines and their parents. J. Agr. Food Chem..

[46] Bohn T (2019). Beta-carotene in the human body: Metabolic bioactivation pathways—From digestion to tissue distribution and excretion. P. Nutr. Soc..

[47] Keys A (2017). The diet and 15-year death rate in the seven countries study. Am. J. Epidemiol..

[48] Wu JHY, Micha R, Mozaffarian D (2019). Dietary fats and cardiometabolic disease: Mechanisms and effects on risk factors and outcomes. Nat. Rev. Cardiol..

[49] Chaudhary V, Bhatia RS, Ahlawat AK (2014). A novel self-organizing map (SOM) learning algorithm with nearest and farthest neurons. Alex. Eng. J..

[50] Gámez Albán HM, Orejuela Cabrera JP, Salas Achipiz OA, Bravobastidas JJ (2016). Aplicación de mapas de Kohonen para la priorización de zonas de mercado: uma aproximación práctica. Rev. EIA..

[51] Kohonen T (2014). MATLAB Implementations and Applications of the Self-Organizing Map.

[52] Martins FA, Carneiro PCS, Da Silva DJ, Cruz CD, Carneiro JES (2011). Integração de dados em estudo de diversidade genética de tomateiro. Pesqui. Agropecu. Bras..

[53] Hu J, Zhu J, Xu HM (2000). Methods of constructing core collections by stepwise clustering with three sampling strategies based on the genotypic values of crops. Theor. Appl. Genet..

[54] Van Hintum TJL, Brown AHD, Spillane C, Hodgkin T (2000). Core Collection of Plant Genetic Resources.

[55] Frankel, O. H. Genetic perspectives of germplasm conservation. In *Genetic manipulation: impact on man and society* (ed. Arber, W. K., Llimensee, K., Peacok, W.J., Starlinger, P.). 161–170 (Cambridge University, Cambridge, 1984).

[56] Malosetti M, Abadie T (2001). Sampling strategy to develop a core collection of Uruguayan maize landraces based on morphological traits. Gent. Resour. Crop Ev..

[57] Moura, M. C. C. L. Identificação de fontes de resistência ao Potyvirus ZYMV e diversidade genética e eco geográfica em acessos de abobora. D. Sc. Thesis, Universidade Federal de Viçosa. 2003. Available from: https://locus.ufv.br//handle/123456789/10265.

[58] Reby D, Lek S, Dimopoulos I (1997). Artificial neural networks as a classification method in the behavioral sciences. Behav. Proces..

[59] Cruz, C. D. Ferreira, F. M. & Pessoni, L. A. Biometria aplicada ao estudo da diversidade genética (Suprema, Visconde do Rio Branco, 2011).

[60] Laurindo RDF, Laurindo BS, Delazari FT, Carneiro PCS, Silva DJH (2017). Potencial de híbridos e populações segregantes de abóbora para teor de óleo nas sementes e plantas com crescimento do tipo moita. Rev. Ceres..

[61] Men X (2021). Physicochemical, nutritional and functional properties of *Cucurbita moschata*. Food Sci. Biotech..

