# Supplementary material for: Artificial neural networks optimize the establishment of a Brazilian germplasm core collection of winter squash (Cucurbita moschata D.)
Source: Sci Rep. 2024 Mar 11;14:5930. doi: 10.1038/s41598-024-54818-y (PMC10928206; doi:10.1038/s41598-024-54818-y)
Supplement: Supplementary file 1 — Supplementary Information. [file 41598_2024_54818_MOESM1_ESM.docx]

1. **Raw data**


Legend of abbreviations
