# Supplementary material for: Artificial neural networks optimize the establishment of a Brazilian germplasm core collection of winter squash (Cucurbita moschata D.)
Source: Sci Rep. 2024 Mar 11;14:5930. doi: 10.1038/s41598-024-54818-y (PMC10928206; doi:10.1038/s41598-024-54818-y)
Supplement: Supplementary file 2 — Supplementary Tables. [file 41598_2024_54818_MOESM2_ESM.docx]

1. **Supplementary Tables**

**Supplementary Table 1**. Detailed origin of part of the *C. moschata* accessions kept in the Vegetable Germplasm Bank of the Federal University of Viçosa

| **Accessions** | **Place of collection** | **Year of**  **collection** | **Accessions** | **Place of collection** | **Year of**  **collection** |
| --- | --- | --- | --- | --- | --- |
| BGH-7219A | Ponta Grossa- PR | 2003 | BGH-5361A | Bocaiuva- MG | 1981 |
| BGH-7668 | Ponta Grossa- PR | 2003 | BGH-5247A | Indaiabira-MG | 1975 |
| BGH-1461A | Planalto Catarinense- SC | 1990 | BGH-6115 | Viçosa-MG | 1985 |
| BGH-6749 | Planalto Catarinense- SC | 1990 | BGH-1004 | Viçosa-MG | 1985 |
| BGH-5472A | Piracicaba- SP | 1984 | BGH-4516 | Indaiabira-MG | 1974 |
| BGH-5541 | Piracicaba- SP | 1984 | BGH-5248 | Indaiabira-MG | 1975 |
| BGH-5556A | Piracicaba- SP | 1984 | BGH-5648 | Juiz de Fora- MG | 1984 |
| BGH-5548A | Piracicaba- SP | 1984 | BGH-5659A | Rio Pardo- MG | 1984 |
| BGH-5453A | Piracicaba- SP | 1984 | BGH-4453 | Viçosa -MG | 1968 |
| BGH-5473A | Piracicaba- SP | 1984 | BGH-4607A | Viçosa -MG | 1968 |
| BGH-5544A | Piracicaba- SP | 1984 | BGH-6155 | Viçosa -MG | 1986 |
| BGH-5591A | Piracicaba- SP | 1984 | BGH-4287A | Aimorés-MG | 1974 |
| BGH-5593 | Piracicaba- SP | 1984 | BGH-4598A | Indaiabira-MG | 1974 |
| BGH-5596A | Piracicaba- SP | 1984 | BGH-5224A | Rio Branco- MG | 1975 |
| BGH-5440A | Piracicaba- SP | 1984 | BGH-6117A | Viçosa -MG | 1985 |
| BGH-5485A | Piracicaba- SP | 1984 | BGH-305A | Viçosa -MG | 1967 |
| BGH-5455A | Piracicaba- SP | 1984 | BGH-3333A | Petrópolis- RJ | 1969 |
| BGH-5598A | Piracicaba- SP | 1984 | BGH-291 | Guanabara- RJ | 1966 |
| BGH-5493A | Piracicaba- SP | 1984 | BGH-5051 | Pirapora- MG | 1975 |
| BGH-5494A | Piracicaba- SP | 1984 | BGH-1961 | Santa Leopoldina- ES | 1967 |
| BGH-5559A | Piracicaba- SP | 1984 | BGH-1945A | Colatina- ES | 1967 |
| BGH-5499A | Piracicaba- SP | 1984 | BGH-1992 | Santa Leopoldina- ES | 1967 |
| BGH-5530A | Piracicaba- SP | 1984 | BGH-5616A | Brasília- DF | 1984 |
| BGH-5606A | Piracicaba- SP | 1984 | BGH-5630A | Brasília- DF | 1984 |
| BGH-5442 | Piracicaba- SP | 1984 | BGH-5624A | Brasília- DF | 1984 |
| BGH-5538 | Piracicaba- SP | 1984 | BGH-315 | Brasília- DF | 1966 |
| BGH-5554A | Piracicaba- SP | 1984 | BGH-5638 | Brasília- DF | 1984 |
| BGH-5301 | Piracicaba- SP | 1984 | GBH-5694 | Brasília- DF | 1966 |
| BGH-5451 | Piracicaba- SP | 1984 | BGH-5639 | Brasília- DF | 1984 |
| BGH-5528 | Piracicaba- SP | 1984 | BGH-6590 | Goiânia- GO | 1988 |
| BGH-5551 | Piracicaba- SP | 1984 | BGH-6587A | Goiânia- GO | 1988 |
| BGH-5552 | Piracicaba- SP | 1984 | BGH-6595 | Goiânia- GO | 1988 |
| BGH-5553 | Piracicaba- SP | 1984 | BGH-6593 | Goiânia- GO | 1988 |
| BGH-5560A | Piracicaba- SP | 1984 | BGH-6794 | Goiânia- GO | 1988 |
| BGH-5597 | Piracicaba- SP | 1984 | BGH-6594 | Goiânia- GO | 1988 |
| BGH-900 | Campinas-SP | 1966 | BGH-6099 | Mossoró- RN | 1985 |
| BGH-5497 | Piracicaba- SP | 1984 | BGH-6096 | Açu- RN | 1985 |
| BGH-5603 | Piracicaba- SP | 1984 | BGH-5653 | Mortugaba- BA | 1984 |
| BGH-5466 | Piracicaba- SP | 1984 | BGH-117 | Salvador- BA | 1966 |
| BGH-5456A | Piracicaba- SP | 1984 | BGH-1749 | Salvador- BA | 1966 |
| BGH-4459A | Viçosa-MG | 1968 | BGH-95 | Feira de Santana- BA | 1966 |
| BGH-4281 | Aimorés-MG | 1968 | BGH-5649A | Mortugaba- BA | 1984 |
| BGH-4454A | Viçosa-MG | 1968 | BGH-5240 | Mortugaba- BA | 1975 |
| BGH-6116 | Viçosa-MG | 1985 | Jabras* | - | - |
| BGH-4590A | Viçosa-MG | 1985 | Tetsukabuto* | - | - |
| BGH-1927 | Aimorés-MG | 1967 | Jacarezinho* | - | - |
| BGH-4681A | Viçosa-MG | 1985 | Maranhão* | - | - |
| BGH-4610A | Viçosa-MG | 1967 |  |  |  |

BGH- Vegetable Germplasm Bank of the Federal University of Viçosa (BGH). The two letters associated with the place of collection refer to Brazilian states where the accession was collected, namely Paraná (PR), Santa Catarina (SC), São Paulo (SP), Minas Gerais (MG), Rio de Janeiro (RJ), Espírito Santo (ES), Distrito Federal (DF), Goiás (GO), Rio Grande do Norte (RN), and Bahia (BA). * These genotypes are commercial cultivars widely cultivated in Brazil.

**Supplementary Table 2.** Descriptors used in agro-mophological evaluation of the 91 accessions of accessions of *C. moschata*

| **Traits** | **Descriptors** |
| --- | --- |
| Reproductive | Accumulated degree-days for flowering (DDF). |
| Fruit | Number of fruits per plant (NFP), average mass of fruits (MF), productivity of fruits (PF), height of fruit (HF), diameter of fruit (DF), thickness of fruit pulp (PT), diameter of internal cavity of fruit (DIC), total content of fruit pulp carotenoids (TC), and the lutein content of fruit pulp (L). |
| Seed | Number of seeds per plant (NSF), mass of seeds per fruit (MSF), ratio of seed to fruit mass (MS/F), mass of one hundred seeds (MOH), productivity of seeds (PS), and seed thickness (ST). |
| Seed oil | Seed oil content (SOC), seed oil productivity (SOP), oleic acid (OA) content, linoleic acid (LA) content, linolenic acid (LNA) content, OA:LA ratio, and polyunsaturated fatty acid (PUFA). |

| **Supplementary Table 3.** *Multi-categorical* descriptors used in agromophological evaluation of the 91 accessions of C. moschata | |
| --- | --- |
| **Traits** | **Descriptors** |
| **Vegetative** | Growth habit, stem colour, intensity of leaf green, leaf silvering, intensity of leaf silvering, leaf serration, presence of trichomes in the leaves, amount of trichomes in the adaxial surface of leaves, amount of trichomes in the abaxial surface of leaves, and leaf recess. |
| **Fruit** | Format of fruits, format of peduncle, number of colours of fruit peel, topography of fruit surface, format of floral scar, peel texture, predominant colour of fruit peel, and depth of fruits slices. |
| **Seed** | Seed format, seed tegument texture, colour of seed tegument, and colour of seed border. |
